# 3D Participatory Sensing with Low-Cost Mobile Devices for Crop Height Assessment – A Comparison with Terrestrial Laser Scanning Data

**DOI:** 10.1371/journal.pone.0152839

**Published:** 2016-04-13

**Authors:** Sabrina Marx, Martin Hämmerle, Carolin Klonner, Bernhard Höfle

**Affiliations:** 1 GIScience, Institute of Geography, Heidelberg University, Heidelberg, Germany; 2 Heidelberg Center for the Environment (HCE), Heidelberg University, Heidelberg, Germany; US Naval Reseach Laboratory, UNITED STATES

## Abstract

The integration of local agricultural knowledge deepens the understanding of complex phenomena such as the association between climate variability, crop yields and undernutrition. Participatory Sensing (PS) is a concept which enables laymen to easily gather geodata with standard low-cost mobile devices, offering new and efficient opportunities for agricultural monitoring. This study presents a methodological approach for crop height assessment based on PS. In-field crop height variations of a maize field in Heidelberg, Germany, are gathered with smartphones and handheld GPS devices by 19 participants. The comparison of crop height values measured by the participants to reference data based on terrestrial laser scanning (TLS) results in R^2^ = 0.63 for the handheld GPS devices and R^2^ = 0.24 for the smartphone-based approach. RMSE for the comparison between crop height models (CHM) derived from PS and TLS data is 10.45 cm (GPS devices) and 14.69 cm (smartphones). Furthermore, the results indicate that incorporating participants’ cognitive abilities in the data collection process potentially improves the quality data captured with the PS approach. The proposed PS methods serve as a fundament to collect agricultural parameters on field-level by incorporating local people. Combined with other methods such as remote sensing, PS opens new perspectives to support agricultural development.

## Introduction

The need for three-dimensional (3D) geodata is rapidly increasing: Detailed geographic information is of critical importance, for example, to understand associations between climate variability, crop yields, and undernutrition [1]. At the same time, a growing number of people own mobile devices equipped with geolocation-aware mobile applications (apps), so that an increasing quantity of geodata is available and, subsequently, cost and knowledge intensive high-end equipment is no longer the only data source. In using the capabilities of every-day mobile computing devices (smartphones or tablets), Participatory Sensing (PS) enables non-professionals to gather geodata in an affordable way without necessarily being GIS experts [2, 3].

By examining also the third dimension, 3D Participatory Sensing offers new opportunities for tackling issues related to food security. For example, crop height information serves as a feedback on the crop health and, thus, is a valuable input for crop failure forecasting [4]. Given the volatility of food production and the effects of climate change, such information is of particular importance for most countries in Sub-Saharan Africa and South-Central Asia [1, 5, 6]: Around 850 million people were suffering from undernutrition between 2010 and 2012 [7]. USAID [8] suggests that mobile computing devices such as smartphones will be increasingly used in data collection efforts for agricultural development. According to Goma *et al*. [9] (p. 178), “participatory processes generate traditional knowledge that is broader and more descriptive than scientific information”. While methods for managing crop production in a spatial and precise manner, known as precision agriculture, have been widely adopted in developed countries, it is still associated with a need for high-end technological equipment [10]. Smallholder farmers know their land best, but this information is mostly neither recorded nor shared. Galindo *et al*. [10] suggest to adopt precision agriculture principles for site-specific management but in a low technology context for such farmers.

However, acquiring agricultural geodata is not trivial especially in low-resource settings, where most farmers grow subsistence crops on small-based plots of land (less than two hectare) [11]. So far smartphones and tablets with built-in low-cost Global Positioning System (GPS) receivers have mostly been used to collect 2D geodata [12]. This study adds the third dimension to agricultural geodata by evaluating in-field crop height variations captured by participants with no experience in geodata collection. Adapting the PS approach to the field of agriculture offers new opportunities for generating data at a local perspective and scale, supplementing data sources such as remote sensing imagery [13]. In PS-based data collection campaigns, participant engagement can range from simply carrying a mobile sensor which is automatically collecting coordinates, to actively contributing their knowledge within the collection process [14]. Both the most basic level and higher levels of participation have been applied successfully in other PS applications [15–18].

This work presents a novel methodological approach for crop height assessments by employing the PS approach. Thereby, this study attempts to utilize the advantages of both, state-of-the-art machine learning techniques and engagement of non-experts to provide new ways for gathering 3D geodata in low-resource settings. Different simple and rapid methods for crop height assessment are developed, and evaluated within a field experiment in which 19 participants are instructed to collect crop heights within a maize field near Heidelberg, Germany. Furthermore, it is analyzed if PS-based crop height assessment benefits from a higher level of participation. In order to assess the quality range of PS data and to predict future usage possibilities of those datasets, gathered crop heights are benchmarked against a highly accurate reference model obtained with terrestrial laser scanning (TLS). Providing low-cost participation methods for agricultural monitoring at field-level is an important contribution to grassroots developments as well as a much-needed source of information for facing challenges such as the effect of climate change on food production of smallholder farmers.

## Previous Work

Participatory Sensing is used in several research fields such as Public Health [15, 16, 19], disaster management [17, 20] and spatial planning [18]. Furthermore, there are several ways in which mobile computing devices can support agricultural development [21]: PS is mainly employed for in-situ data collection, but it also showed high potential for improving agricultural awareness and education activities [22]. In addition, PS is a beneficial tool for plant disease monitoring [23]. Kotovirta *et al*. [24] conduct a pilot study to collect information about the disease situation at nine farms in southern and central parts of Finland using the *EnviObserver* framework. *EnviObserver* [25] (p. 1) is a set of tools “that utilizes people as living sensors, by enabling reporting of environmental observations using a mobile phone”. Several other platforms and tool sets are used to collect geographic information with mobile phones such as *EpiCollect* [26] or the *OpenDataKit* (*ODK*) [27]. For example, Enenkel *et al*. [28] present the mobile app *SATIDA COLLECT* for food security monitoring in the Central African Republic which is build using the *ODK* toolkit. Moreover, several smartphone/tablet apps have been developed to collect agricultural parameters such as the *Nitrogen Index* app [29] or an app for estimating leaf area index [30].

For gathering geographic information with smartphones different methods are employed (e.g. geotagging or recording GPS trajectories). Geotagging means the annotation of media or information such as text messages, images or videos with geographic coordinates [31]. It is, for example, used within the *CalFora* project [32], in which participants are asked to collect plant observations (including plant name, photo, point location etc.). Higuera *et al*. [33] use smartphones to record GPS trajectories of moving vehicles for road inventories. A further method of using smartphone-based systems for mapping small objects such as crop fields or fish ponds is suggested by Schmid *et al*. [34]. The developed *MapIT* system allows capturing small geographic objects with high geometric accuracy [35]. The user takes a photo of the object of interest and draws the outline on the phone’s screen. Finally, the extracted geometry is transformed into a geo-object [34]. Extracting information from images provides new opportunities for smartphone-based data collection campaigns. Heipke [36] (p. 556) suggests that “future mapping may only involve taking images of objects of interest; turning the image into meaningful information is then accomplished employing real-time automatic image interpretation, perhaps supported by the photographer providing a written annotation”. Furthermore, Ferster *et al*. [13] point out the potential of incorporating judgment-based information collected by participants in an automatic classification of photographs.

These examples show a wide range of possible applications for PS, but also the limitations of this approach. Although PS provides the means to collect valuable geographic information, to ensure high quality of so-called Volunteered Geographic Information (VGI) is pointed out as one of the major challenges (e.g. [37, 38, 39]): For example, there may be a low accuracy of GPS positions captured with low-cost GPS units and of collected attributes. The latter can be caused by users, for example, if participants misunderstand instructions given to them, lack expertise or mistakenly submit erroneous data or by errors in the recorded data, for example, geo-tagged pictures that are too dark or blurry to extract any information [13, 40]. Furthermore, PS applications have been mostly used to collect 2D geodata; a PS-based approach for crop height assessment on a scale of individual crop field plots is missing. Investigating the potential of PS for 3D geodata collection is not only beneficial for facing the challenges in the context of agricultural development, it can also be adapted to other research fields e.g. disaster management to map flood water levels in urban areas [41].

## Study Area and Sensors

**Fig 1:**
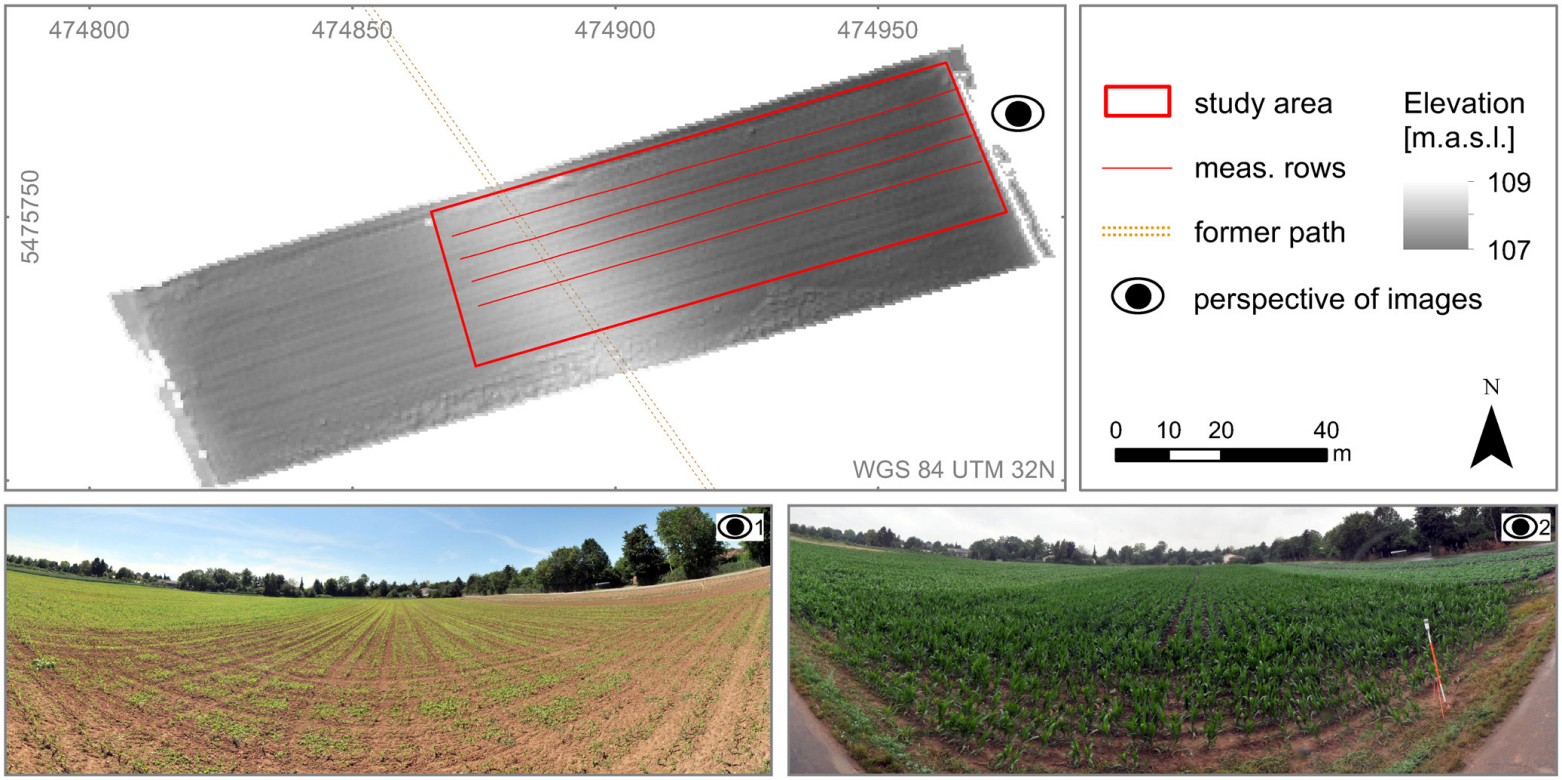
Study area located on a maize field in Heidelberg, Germany (north-eastern Baden-Wuerttemberg). The location of the ancient Roman street (former path) as well as the four predefined measurements rows are shown. The underlying DTM is colored by elevation above sea level. Perspective 1: panorama image taken at the eastern end of the study area on 2014/06/06. Perspective 2: panorama image taken on 2014/07/08.

The study site is a maize field (150 m × 30 m) located in the Handschuhsheimer Feld in Heidelberg, Germany (49°25'57"N, 8°39'29"E). The study area is shown in Fig 1. Corn was sowed with about 15 plants per m^2^ and about 15 cm distance between each plant in a row, row spacing is about 80 cm. The field is mostly flat but comprises minor depressions, and the elevation slightly increases towards the field due to remainders of an old, probably Roman, street crossing the area [42]. The former path, nowadays buried under soil, additionally influences plant growth, so that subsequently heights are about 40 cm lower (July 8, 2014) in this area compared to the surrounding plants.

The PS activities are conducted in the second week of July 2014 (07/07, 07/09 and 07/11). Weather is cloudy at the beginning of the week, drizzly on July 9 and sunny at the end of the week. The group of volunteering participants consists of 19 undergraduate geography students who collect crop height measurements along four predefined maize rows for four hours. Two out of the 19 participants have experiences with geocaching [43], the other participants have never actively gathered geographic information or used a GPS device before. To compare two low-cost sensors, crop heights are collected using i) smartphones as well as ii) handheld GPS devices. Therefore, the participants are separated randomly in two groups. One group is equipped with six Garmin Oregon 550 devices (typical 2D accuracy of 10 m at a 95% confidence level, [44]), the other group uses smartphones for gathering crop height information In sum, nine different smartphone models from four companies (Samsung, HTC, Motorola and LG) are used whereby the built-in GPS receivers as well as the cameras are employed. GPS-enabled smartphones can achieve a horizontal positional error of several meters [45]. Overall, GPS measurements with handheld GPS devices are mostly of higher positional accuracy compared to common smartphones and tablets [46]. The quality of the built-in cameras ranges from five megapixels (e.g. Samsung S III mini, Samsung Giorgio Armani Galaxy S, Motorola Moto G) up to eight megapixels (Google Nexus 4, Samsung Note 3 neo, HTC sensation).

Reference data is collected by a Riegl VZ-400 TLS during two field surveys eight weeks apart. Several articles evaluate laser scanning for deriving crop parameters [47–53]: Detailed information about crop height and crop stand geometry is obtained by laser scanning, thus, justifying it as a suitable reference for comparison with PS data. During the first campaign, data for generating a Digital Terrain Model (DTM) is acquired, which represents the soil surface without objects (i.e. plants); while during the second field survey data for a Crop Surface Model (CSM) representing the surface of the crop stand is collected. Subsequently, a Crop Height Model (CHM) is generated by subtracting the DTM from the CSM. The first TLS campaign is performed on June 6, 2014 when most of the plants are between 10 cm and 20 cm high and the bare ground can still be captured by TLS without occlusion by the plants. One month later on July 8, 2014 the second TLS survey takes place for capturing the crop surface.

The Riegl VZ-400 measures the distance between sensor and object according to the time-of-flight principle [54]. 3D coordinates (x, y, z) of the measured object points are calculated. Additionally, a digital camera is mounted on top of the laser scanner to record RGB values which are assigned to the measured 3D coordinates. Within the first scan campaign in June, high-resolution point clouds are acquired from six scan positions with a point spacing of 5 mm at 10 m distance. The second campaign in July provides data from seven scan positions with the same point spacing.

## Methodology

**Fig 2:**
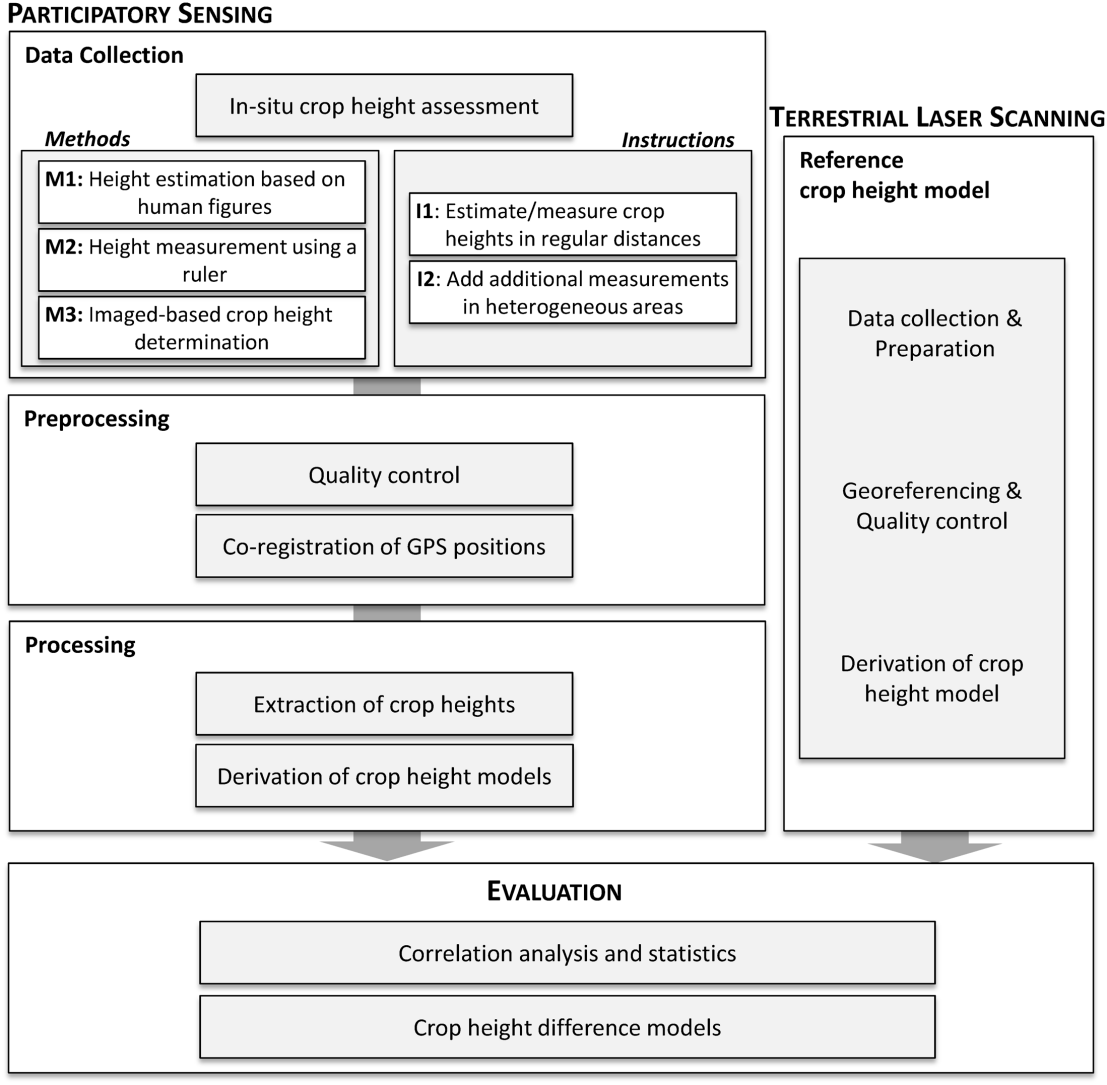
Workflow diagram showing the main processing steps from data collection to evaluation. The corresponding sub steps are shown in the grey shaded boxes.

The workflow shown in Fig 2 is developed in order to assess the potential of PS for gathering 3D crop height information. In the first step, a field experiment is designed and conducted in order to gather the in-field crop height variations using different data collection methods and instructions. The field experiment of this study was announced in a GIScience introductory course at Heidelberg University. The voluntarily participating students all knew that they will collect data on plant heights for research purposes and gave their consent. A study-specific ethics approval was not needed as only non-sensitive data was recorded. The study was carried out on private land; permission to conduct the field experiment was given by the owner. No further specific permission was required as only non-invasive and non-destructive methods were applied for plant height assessment. After in-situ data collection, data are preprocessed, including georeferencing the TLS data and the co-registration between the collected GPS positions and the TLS dataset. In the next step, crop height information is extracted from the gathered PS dataset and analyzed (anonymously). Finally, the crop height measurements are compared to the reference crop height model derived from TLS. In the following, the mentioned steps are explained in detail.

### Participatory sensing data collection

Crop height is assessed using three different methods: directly by the participants, using (M1) human figures to estimate as well as (M2) a ruler to measure heights, and (M3) automatically from photographs using a supervised machine learning technique. Furthermore, the effect of participation level is analyzed by giving two different types of instructions to the participants for collecting in-field crop height variations: The first instruction (I1) requires minimal engagement of the participants’ cognitive ability (Participatory Level 1, [14]), whereas the second instruction (I2) is based on a higher level of participation (Participatory Level 2, [14]). In order to compare the smartphone-based approach (SP group) with data collection using handheld GPS devices (GPS group), participants are randomly separated into two groups. For the smartphone-based approach, a mobile application is built using the *ODK* toolkit whereas paper-based data collection forms (similar to the digital collection forms) are handed out to the GPS group. The *ODK* is a free and open-source toolkit which is suitable to support mobile data collection in developing countries [55, 56]. For example, Rajput *et al*. [57] successfully used the *ODK Collect* app for population surveillance in Kenya. In our study, *ODK Collect* V.1.4.4., supporting several data types for data collection such as text, images as well as GPS locations, has been installed on the participants’ smartphones. The finalized data collection forms are uploaded to the *ODK Aggregate* module which provides a server for data upload, storage and transfer [27].

#### Instructions

General instructions about how to capture coordinates using GPS devices and smartphones are given for half an hour at the beginning of the data collection session since most of the participants have never captured geographic information before. As each applied data collection method (M1-M3) has its own specific requirements, a ten-minute oral instruction is given before a new collection round starts. To guide participants during the data collection process, a step-by-step instruction is also integrated into the *ODK Collect* app (S1 Table). A handout containing these steps is distributed to the GPS group. Furthermore, method-specific instructions are provided which operate on different engagement levels: For direct crop height assessment, the participants are asked to capture GPS positions and estimate as well as measure crop heights every tenth step in four predefined rows (I1) and add additional measurement positions in between if plants vary greatly in height (I2). In the case of I2, positions for height assessment are selected on subjective judgment of the participants, whereas I1 relies on a lower level of participation. Thus, data collection follows a regular and therefore more controlled pattern. To avoid that participants are influenced by other participants collecting the same parameter nearby, participants start data collection from different starting positions.

#### Direct crop height measurements

**Fig 3:**
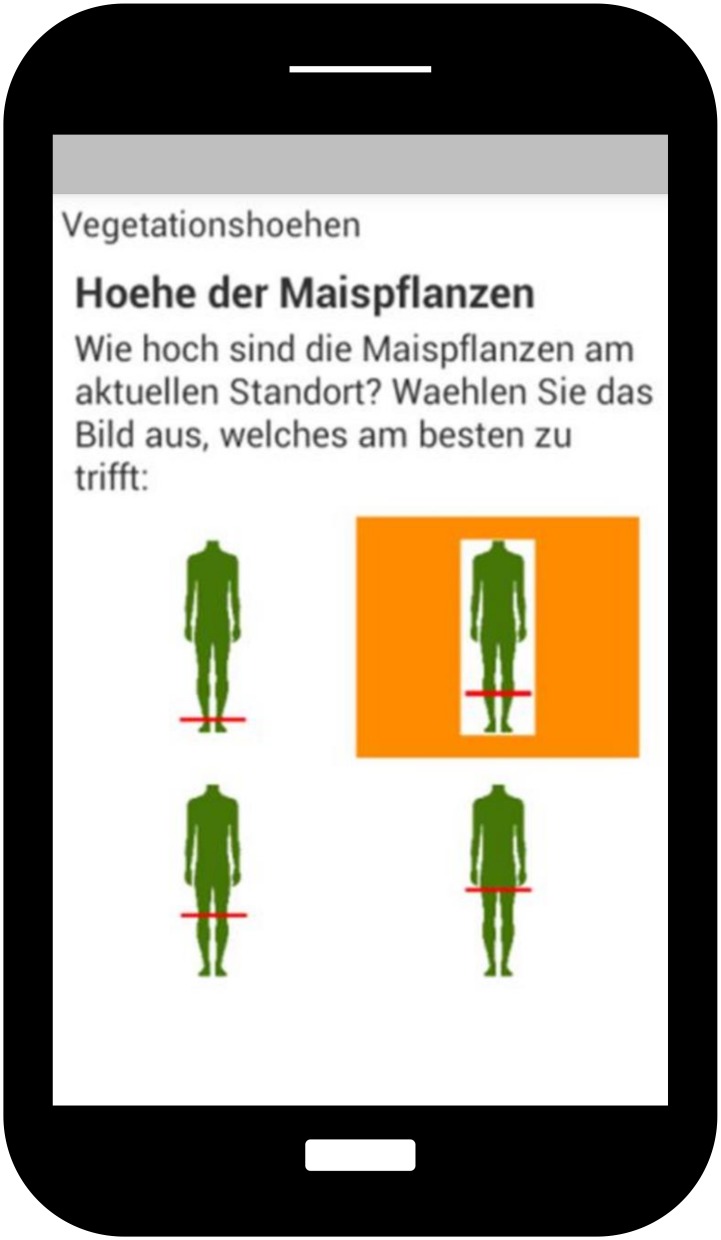
Selected screenshot of the *ODK Collect* app (data collection method 1: height estimation based on human figures). Translation of the instructions displayed on the smartphone’s screen in German: “Maize plant height—How high are the maize plants at the current position? Please choose the image which fits best:”.

Direct crop height assessment within the maize field is based on two different methods: For each obtained GPS position, the height of the corresponding plant is measured by the participants using a ruler (M2). Furthermore, plant heights are estimated by choosing one out of ten symbols showing a human figure marked at ten different height levels e.g. knee or head (M1). The figures are presented to the participants either on their smartphone displays (Fig 3) or on a paper according to their group. A similar method is successfully used by Degrossi *et al*. [41] to build up a Flood Citizen Observatory. To assign height values to each height interval, body height as well as knee height is entered by each participant at the beginning of the survey. The measurements of crop heights using a pocket ruler (M2) are done at the same position where heights were estimated beforehand. Thereby, participants are asked to measure the top of the highest leaf of the maize plant. GPS location is added to each measurement position using smartphones and handheld GPS devices respectively. To achieve a dense measuring network, the direct height measurements are captured in four predefined measurement rows.

#### Image-based crop height assessment

Image processing is another way to gather relevant information about geographic objects. Thus, height information is not directly assessed by a human being, but is indirectly determined from images using an automatic image processing technique (M3): The participants take a picture of the maize field in which a marker bar is placed to provide a height reference. Thereby, the smartphone camera is located above the plants focusing the marker bar. After taking the images, participants are asked to draw a circle around the marker bar on the touchscreen of their smartphones. This circle is used to support the automatic classification of the photographs (see Section 4.2.2. for further details). Furthermore, GPS coordinates are measured for every position where the images are taken from. This method is only performed by the SP group.

### Processing of participatory sensing data

The steps conducted to extract crop height information from the gathered PS geodata are described in the following. In Section 4.2.1. the methods for crop height determination based on the direct approach (M1&M2) are introduced, followed by details on the image-based method (M3) in Section 4.2.2.

#### Direct crop height measurements

Crop height extraction based on the direct measurements is straightforward. For each GPS position the estimated height values as well as the ruler-based measurements are gathered. The estimated height values are collected in ten different categories based on the human figures marked at equal height intervals. Thus, the categories have to be converted into a metric system. This is done via the body height which is recorded by the participants representing the largest height category.

For benchmarking the PS height measurements with the reference TLS crop heights, co-registration of the captured GPS positions to the TLS data is required. The overall distance between the GPS coordinates and the four pre-defined measurement rows extracted from the TLS dataset is minimized using a similarity transformation which maintains the relative shape of the GPS measurements. The similarity transformation only scales, rotates or shifts the data but does not skew the GPS measurements [58].

In the next step, an area-wide crop height model for the study area is derived by interpolating the point-based crop height measurements (named CHMPS in the following). Spatial interpolation of PS data is addressed by Mendez *et al*. [59]. The authors propose Kriging as an appropriate interpolation for PS measurements system. Kriging exploits the statistical relationship among the points in a spatial dataset to forecast the process at a certain location [60]. In this study Empirical Bayesian Kriging implemented in the Geostatistical Analyst extension of ArcMap 10.1 is applied to automatically build up a valid Kriging model [61, 62].

#### Image-based crop height assessment

**Fig 4:**
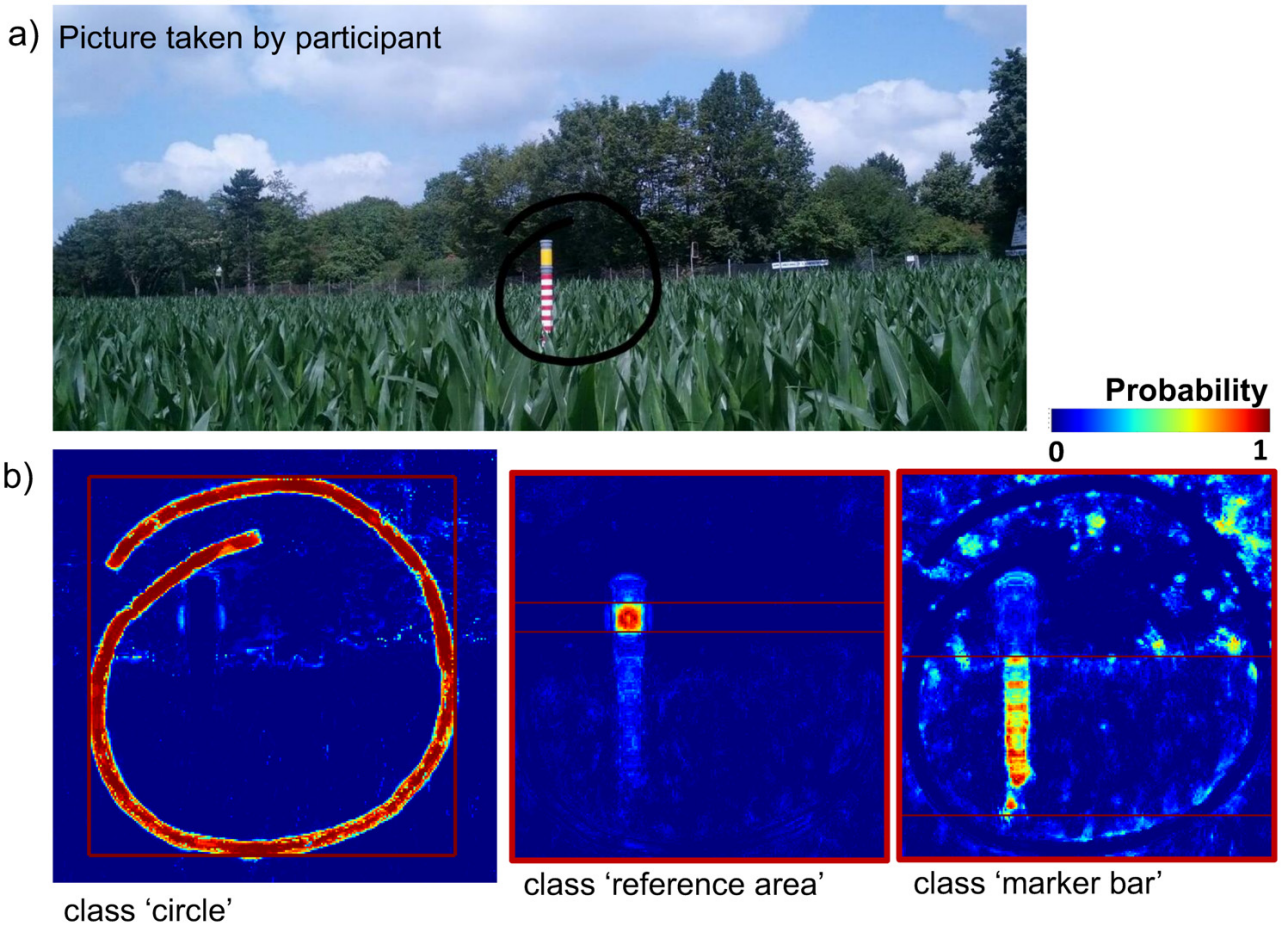
Image-based crop height assessment. (**a**) Picture taken by a participant on July 11, 2014 (published with permission from Johanna Schwehn). The area of interest was marked by drawing a circle around the marker bar; (**b**) Probability maps resulting from the image classification. The vertical extent of the reference area and of the marker bar is shown in the probability maps.

Image processing is used to extract information about crop heights from the photographs (M3). Sritarapipat *et al*. [63] propose a method for automatic rice crop height measurements using a fixed installed marker bar and a field server equipped with a camera. A simple image processing algorithm based on band combinations is used to indirectly measure heights based on the images showing rice plants as well as a marker bar: As the rice grows higher, more parts of the marker bar is obscured [63]. Since in the field experiment presented here pictures are taken from different positions at different times with diverse built-in cameras, a more advanced approach is required to assess the crop heights. In a first step, image segmentation is performed by using supervised machine learning to identify (1) pixels representing the visible part of the marker bar, (2) the reference area, and (3) the manually drawn circle to incorporate the participants’ knowledge (Fig 4a). Second, the region for further processing is clipped to the bounding box of the extracted circle. Third, the height of the visible part of the marker bar is calculated based on the segmentation result and the known height of the reference area. Finally, the height of the visible part of the marker is subtracted from the total height of the marker bar and thus the crop height is extracted.

The free and open-source project Interactive Learning and Segmentation Tool Kit (*ilastik*) is used for pixel-wise classification of the gathered pictures which enables interactive image classification using Random Forests (RF) [64]. The applied workflow for supervised pixel classification consists of three parts: 1) label a randomly selected subset of the images 2) compute additional image features and 3) train a RF classifier and automatically classify the entire dataset. As training dataset, ten percent of the images are randomly selected. These images are manually labeled using the *ilastik* user interface. Image labeling is guided by the interactive training mode in *ilastik* that provides real-time feedback on the current classifier prediction as well as an uncertainty measure (active learning).The classes are: marker bar, reference area, circle and background. In the next step, several image features are computed that characterize the neighborhood of each pixel such as edge indicators as well as color, texture and orientation functions. The results of the pixel classification are probability maps, in which the probability of belonging to one of the predetermined classes is assigned to each pixel (Fig 4b). Thresholding by a trial-and-error process is used to create binary images (probability threshold = 50%) to identify the objects of interest (marker bar, reference area and circle). Next, small misclassified objects in the binary images are removed by using binary opening [65]. After removing noise from the images, binary dilation is applied to connect nearby objects. Then, the largest connected area in each image is identified representing the objects of interest.

Before extracting the marker bar and the reference area, both images are clipped to the bounding box of the classified circle which was added to the pictures by the participants during the data collection process. Thus, only a specific region of the image is considered as being relevant for the extraction of the marker bar. Following Ferster *et al*. [13], this approach aims at avoiding misclassifications due to objects in the background with similar characteristics by incorporating the information from the participants taken in the field. Once the marker bar and the reference area have been identified in the binary images, each pixel column is summed up in vertical direction. The highest pixel number is assigned to the height of the visible part of the marker bar as well as to the reference area in the image. The height of the visible part of the marker bar can be calculated because the actual height of the reference area is known (15 cm). Plant heights are extracted by subtracting the height of the visible part of the marker bar from the total height of the marker bar which is 200 cm.

### TLS reference crop height model

After data collection and preprocessing of the TLS data, a detailed reference CHM representing the uppermost leaves of the maize plants is derived by taking the difference between the TLS-surface model and the DTM. The preprocessing step includes transferring the single scan positions into a common global coordinate system which is done with the proprietary RiSCAN PRO v.1.8.0 software provided by RIEGL [66]. Firstly, the scanner’s position and orientation is automatically determined based on matching tie points. Secondly, corresponding global coordinates are assigned to each tie point. The global coordinates of six tie points used for georeferencing the scans are measured with a Leica TCRA705 power total station. Georeferencing results in 2.3 cm (first campaign) and 2.0 cm (second campaign) standard deviation of residues between the position of tie points determined with the total station and tie points in the registered laser scan. Point density is in average 74 points (first campaign) and 86 points (second campaign) per 0.01 m^2^. Thus, highly-accurate TLS data is available for benchmarking the PS data.

For deriving the DTM, off-terrain points are filtered from the point cloud using the Terrain Filter in RiSCAN PRO [66]. Next, a rasterized DTM from the filtered point cloud is derived using the OPALS Module Grid [67]. Moving planes interpolation is used to map height values from the point cloud to the raster cells. A grid size of 50 cm is chosen based on the derived quality parameters (e.g. point density), which results in a seamless DTM. The derivation of the CHM consists of the following steps: (1) A CSM is generated by extracting the maximum elevation for every grid cell. To exclude possible outliers, the 99^th^ percentile of elevation is mapped to the grid cells [68]. (2) For determining vegetation height, the DTM is subtracted from the CSM. (3) In order to consider the uppermost leaves of each plant only, the local maxima in vegetation height are extracted using a circle-shaped kernel with a diameter of 15 cm. This diameter is chosen with respect to the average plant spacing along the rows (15 cm). Thus, the height of single maize plants is represented in the resulting CHM_max_15cm_. (4) As the average row spacing is about 80 cm, the derived CHM_max_15cm_ does not only represent the height of the uppermost leaf but also of lower maize leaves. Thus, the 15 cm CHM_max_ is filtered in a second processing step: Local height maxima are extracted by using kernel of 80 cm diameter, which is the value for the average row spacing. Based on the assumption that crop heights do not change more than 10 cm within 80 cm distance, firstly, a difference raster between both TLS CHMs (CHM_max_80cm_—CHM_max_15cm_) is calculated. Secondly, grid cells with height difference values higher than 10 cm are filtered out as they are assumed to represent lower plant parts. (5) Finally, a Gaussian filter is applied to derive a seamless CHM_FiltGaus_ based on the CHM_Filt_. The final CHM_FiltGauss_ is named CHMREF in the following.

### Evaluation

The gathered PS crop heights are evaluated via a comparison with the CHMREF using the coefficient of determination (R^2^). As the Pearson correlation coefficient is not robust to outliers [69], data values greater than the 95^th^ percentile of the height differences between the PS and TLS heights are excluded from the correlation analysis. Additionally, crop height difference models CHMREF—CHMPS are derived and basic statistics are calculated (e.g. root-mean-square-error (RMSE), mean, min-max, standard deviation (SD)).

Furthermore, the classification result of the image-based approach (M3) is assessed by calculating precision (*p*) and recall (*r*) (Eqs (1) and (2)) using manually labelled pictures as ground truth. Precision is a measure of exactness, i.e. the percentage of pixels correctly labeled as marker bar, whereas recall represents completeness. These metrics are better suited to the class imbalance problem since the total number of pixels of the main classes (marker bar and reference area) is far less than the number of pixels of the background class.

precision=TP/(TP+FP) (1)

recall=TP/(TP+FN) (2)

In Eqs (1) and (2), TP is the number of true positives, FP the number of false positives and FN the number of false negatives.

## Results and Discussion

The results of creating the CHMREF are described in Section 5.1. Section 5.2. contains the results and discussions of the PS approach as well as the comparison between the extracted PS crop heights and the CHMREF. If not stated otherwise, measured PS crop heights (M2) gathered according to I1 are considered for the evaluation. Further insights with respect to the influence of instruction type as well as data collection method are presented in the sections 5.2.2., 5.2.3. and 5.2.4.

### TLS reference crop height model

In the following, the CHMs are described which are derived from the TLS campaigns. In a preparatory step, 73% of the georeferenced TLS points of the first scan campaign are classified as terrain points using the statistical terrain filter module of RiSCAN PRO. The resulting DTM with a pixel size of 50 cm is based on 42 million ground points. DTM elevations range between about 107 m and 109 m above sea level (a.s.l.). Maximum elevation is found in the center of the field. The CHMREF is derived for the entire study area in which participants are instructed to collect crop height values. In-field crop height variations are clearly visible in this model; heights range from 30 cm up to 180 cm. In the area of the Roman street as well as at the eastern end of the study area, plants are about 40 cm lower compared to the surrounding plants. The highest plants (150 cm to 180 cm) are found at the northern edge of the study area.

### Participatory sensing of crop heights

The PS campaign results in 630 direct crop height measurements. 273 height values are gathered by six participants with the paper-based approach using handheld GPS devices, and 357 measurements are collected by six further participants using smartphones (S1 and S2 Datasets). An overview of the gathered PS dataset with respect to the applied data collection method and instruction is presented in Table 1. Each GPS point is associated with two height values—one is measured with a ruler (M2), the other one is estimated based on the human figures (M1). 83% (GPS group) and 71% (SP group) of the crop heights are gathered based on I1. Furthermore, participants marked 17% (GPS group) and 29% (SP group) of the collected positions as additional measurements according to I2. Capturing one GPS position as well as the corresponding attributes takes 1 minute and 36 seconds on average when handheld GPS devices are used. One smartphone-based height measurement takes about 1 minute and 25 seconds on average (including the time which is needed to walk from one measure point to the next). Automatic crop height extraction (M3) does not require any measurement but participants are asked to take pictures and mark the position of the marker bar in the pictures. In total 81 pictures are taken from the marker bar on all three days of the PS experiment.

**Table 1:** Overview of the georeferenced Participatory Sensing dataset on crop heights gathered within a maize field in Heidelberg.

Method No.	Sensor type	Number of participants	Total number of measurements	Number of meas. based on I1	Number of meas. based on I2
**M1 & M2**	GPS devices	6	273	226	47
**M1 & M2**	Smartphones	6	357	253	104
**M3**	Smartphones	13	81	--	--

Participants upload the finalized questionnaires (M1-3) from the *ODK Collect* app to the *ODK Aggregate* server. This can be done after the in-situ data collection, once there is internet connection available. As soon as the data are uploaded, they are visualized on the *ODK Aggregate* server using maps and simple graphs. Data quality might be improved by real-time feedback about the user’s observation in relation to other observations in the area [24]. Rafoss *et al*. [23] (p. 339) suggest that digital data collection “represents a great untapped potential for efficiency and quality improvement”. However, in developing regions there is still a lack of internet connectivity [5] which is required for a two-way communication. In addition, geodata acquisition is challenged by battery life time and internal memory of the smartphones since data is stored internally prior to submission to the server. Moreover, it has to be considered that smartphones are not designed for use in all outdoor conditions. Rajput *et al*. [57] show that participants find the forms displayed on smartphones difficult to read under direct sunlight or in low light conditions. In this study, participants report that the *ODK Collect* app is easy to use. This is consistent with the findings of Enenkel *et al*. [28] (p. 11) who conclude that *SATIDA COLLECT* (based on *ODK*) “seems to be the most flexible, efficient and user-friendly data collection tool”that their local cooperation partners in the Central African Republic have used so far. Nevertheless, a short training and some support is needed depending on the participant’s skill e.g. to install the apps. In comparison to the paper-based approach using handheld GPS devices, data collection with smartphones is more time-efficient. Attributes collected with the paper-based approach have to be manually digitized and linked to the corresponding GPS positions, which is time-consuming and error-prone.

#### Direct crop height measurements

**Fig 5:**
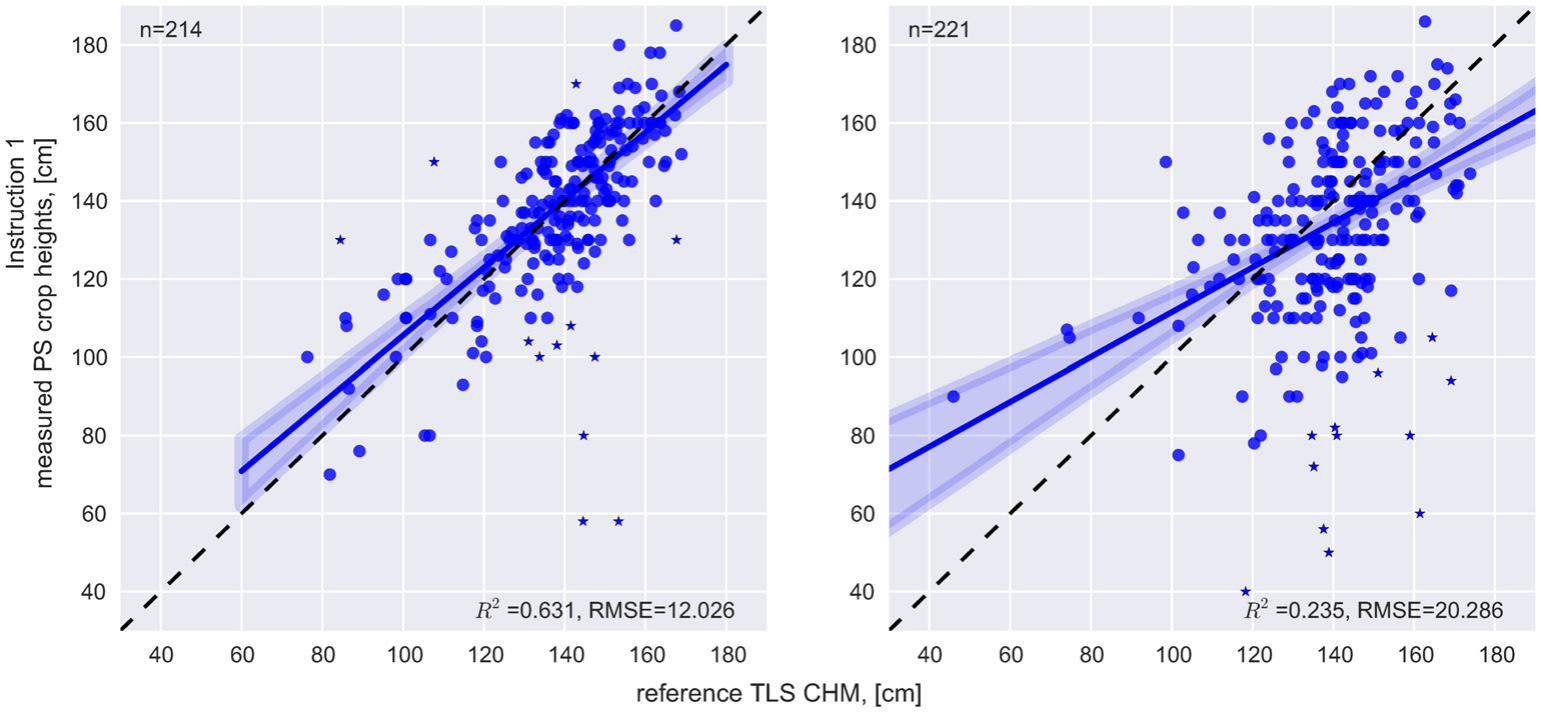
Comparison between PS and TLS crop heights. For the correlation analysis, data points with values higher than the 95^th^ quantile of height differences between TLS and PS crop heights are labelled as outliers (blue stars).

Co-registration between the measured GPS positions and the reference dataset is performed separately for each of the four measurement rows. It results in a RMSE of 5.15 m (smartphones) and 1.19 m (GPS devices) between the predefined rows and the adjusted GPS positions. Correlation analysis shows a moderate (R^2^ = 0.63, GPS group) and weak (R^2^ = 0.24, SP group) relationship between the adjusted PS and the CHMREF (Fig 5). The applicability of manual measurements of crop height is also shown by Tilly *et al*. [53] who report high coefficients of determination between a TLS CHM of a paddy rice field and manually measured crop heights (R^2^: 0.72 to 0.91). However, in contrast to this study, crop heights were measured by the researchers themselves.

**Fig 6:**
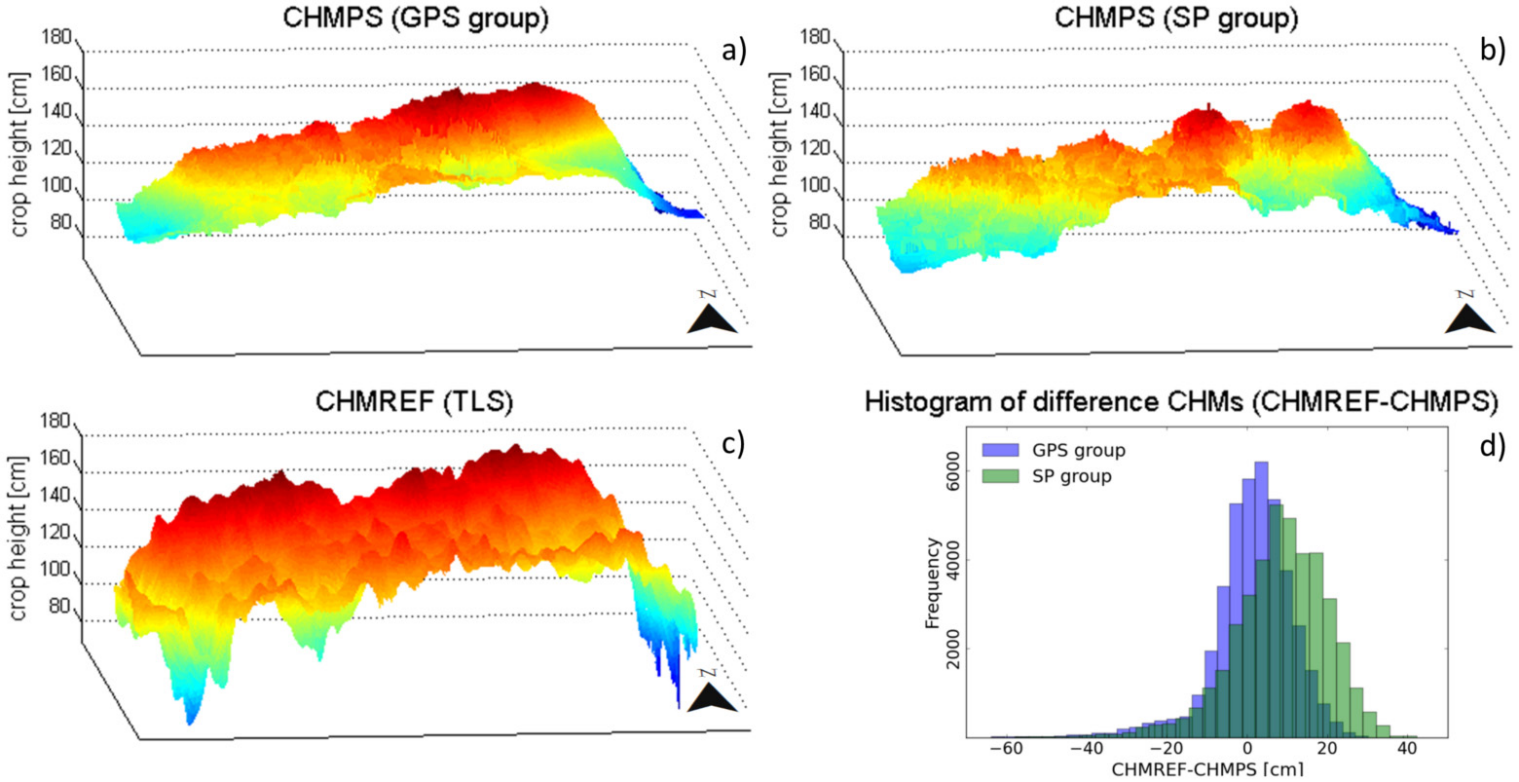
Surface plots showing the in-field crop height variations within the study area. (**a**) Surface plot of the interpolated PS crop height model based on 226 crop height measurements gathered by the GPS group; (**b**) PS crop height model based on 253 crop height measurements gathered by the SP group; (**c**) Reference crop height model derived using terrestrial laser scanning; (**d**) Histogram of difference CHMs (CHMREF-CHMPS) derived for the GPS group as well as for the SP group.

Fig 6a and 6b present 3D surface plots of the CHMPS for the GPS group as well as for the SP group. The general spatial patterns of heights within the maize field (Fig 6c) are visible in both plots: Lower heights at the western and eastern parts of the study area, higher values at the northern boundary. However, the value range of the CHMPSs is smaller i.e. maximum and minimum crop heights are not represented. Overall, PS-based measurements tend to underestimate the absolute crop height as shown in Fig 6d: The average absolute height difference (CHMREF-CHMPS) is 1.35 cm (SD = 10.37 cm) for the GPS group and 8.15 cm (SD = 12.15 cm) for the SP group (mean height of the CHMREF = 137 cm). RMSE of this comparison is 10.45 cm (GPS group) and 14.69 cm (SP group). Reducing the number of PS measurements by applying a minimum distance threshold indicates that the overall spatial height pattern of the crop field is still visible even if only one PS measurement every tenth meter is available.

The higher correspondence for measurements of the GPS group compared to the SP group indicates that the benchmark against the CHMREF is strongly determined by the positional accuracy of the GPS units. Several authors address the positional accuracy of crowdsourced data acquired with low-cost GPS devices [70, 71] and various methods are suggested which help to overcome the identified limitations: Additional information, for example, obtained from multiple built-in sensors of the smartphones (e.g. accelerometers) can be used to improve GPS positioning [72]. Hedgecock *et al*. [73] (p. 221) present another method in which low-cost GPS-receivers in a “network share their raw satellite measurements and use this data to track the relative motions of neighboring nodes”. The authors claim that centimeter-scale tracking accuracy is possible with the suggested method. In addition to technical solutions, incorporation of human’s cognitive abilities provides opportunities to improve positional accuracy. Measured GPS positions, for instance, can be validated during data collection by visualizing them on a smartphone display overlaid by other geographic information [74]. Participants may even be allowed to adjust the measured point in an interactive map [75]. Incorporating the presented methods in the PS data collection process can lead to a better positional accuracy and provide means to increase the quality of the collected PS data.

#### Comparison between estimated and measured crop heights

**Fig 7:**
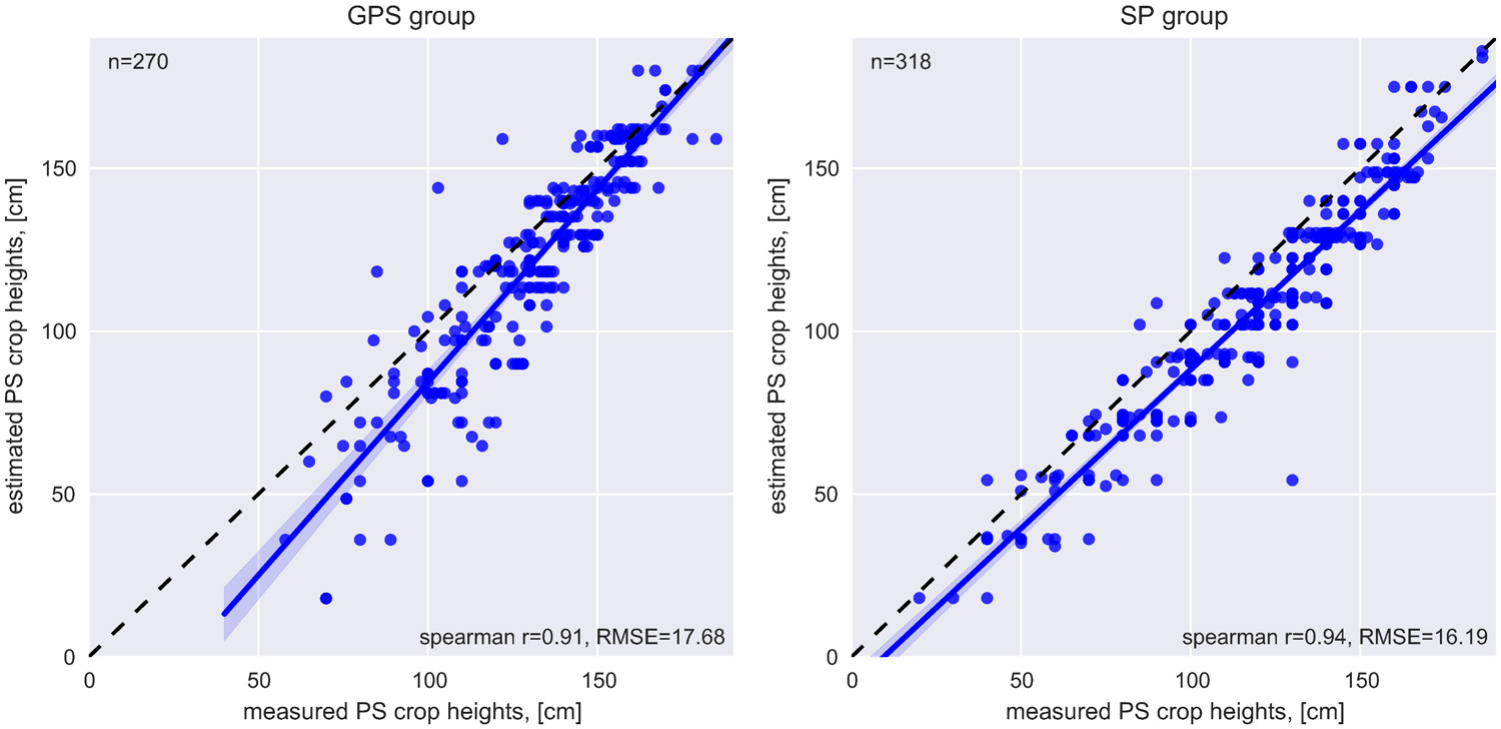
Comparison between estimated crop heights based on human figures (M1) and measured heights using a ruler (M2).

A strong correlation is found between crop height values captured with a ruler (M2) and assessed via human symbol figures (M1). Spearman's correlation coefficient is 0.94 for the smartphone-based data collection and 0.91 for the GPS group. The left plot in Fig 7 shows that for the GPS group deviation between measured and estimated heights is higher for smaller plants (less than 120 cm). This trend is not found in the data collected via smartphones. RMSE is 17.69 cm for the GPS group and 16.19 cm for the SP group; in average underestimation of crop heights is higher if heights are gathered based on M1 compared to M2 (mean crop height for GPS group: 121.88 cm [M1], 131.72 cm [M2]; mean crop height for SP group: 106.07 cm [M1], 118.32 cm [M2]).

One explanation for the differences between the SP and GPS group is the way how the estimated height values are collected: The participants who are using smartphones directly click on the symbol which represents plant height best. In contrast, the human figures are provided on a hand-out for the GPS group. The ID of the symbol which fits best to the actual crop height is written in the paper collection form which is error-prone. For the proposed estimation method based on human figures (M1), no additional equipment is needed which lowers the barriers for participation. The size of height intervals has to be chosen by the experiment designer depending on the desired object size and level of detail. Although crop heights are not measured on a centimeter-scale but are categorized into classes of 15 cm to 20 cm, depending on the body height of the participant, estimating 3D heights based on symbols offers great potential for PS-based crop height assessment. A similar method is used by Degrossi *et al*. [41] for flood citizen observations. An illustration of a human figure is painted on a riverbed of the rivers of São Carlos, Brazil. The measurement of water level is carried out according to pre-defined tags e.g. knee or above the head. The authors conclude that this is an effective way to obtain useful VGI since it can be easily provided by the participants.

#### Comparison between Instruction 1 and Instruction 2

**Fig 8:**
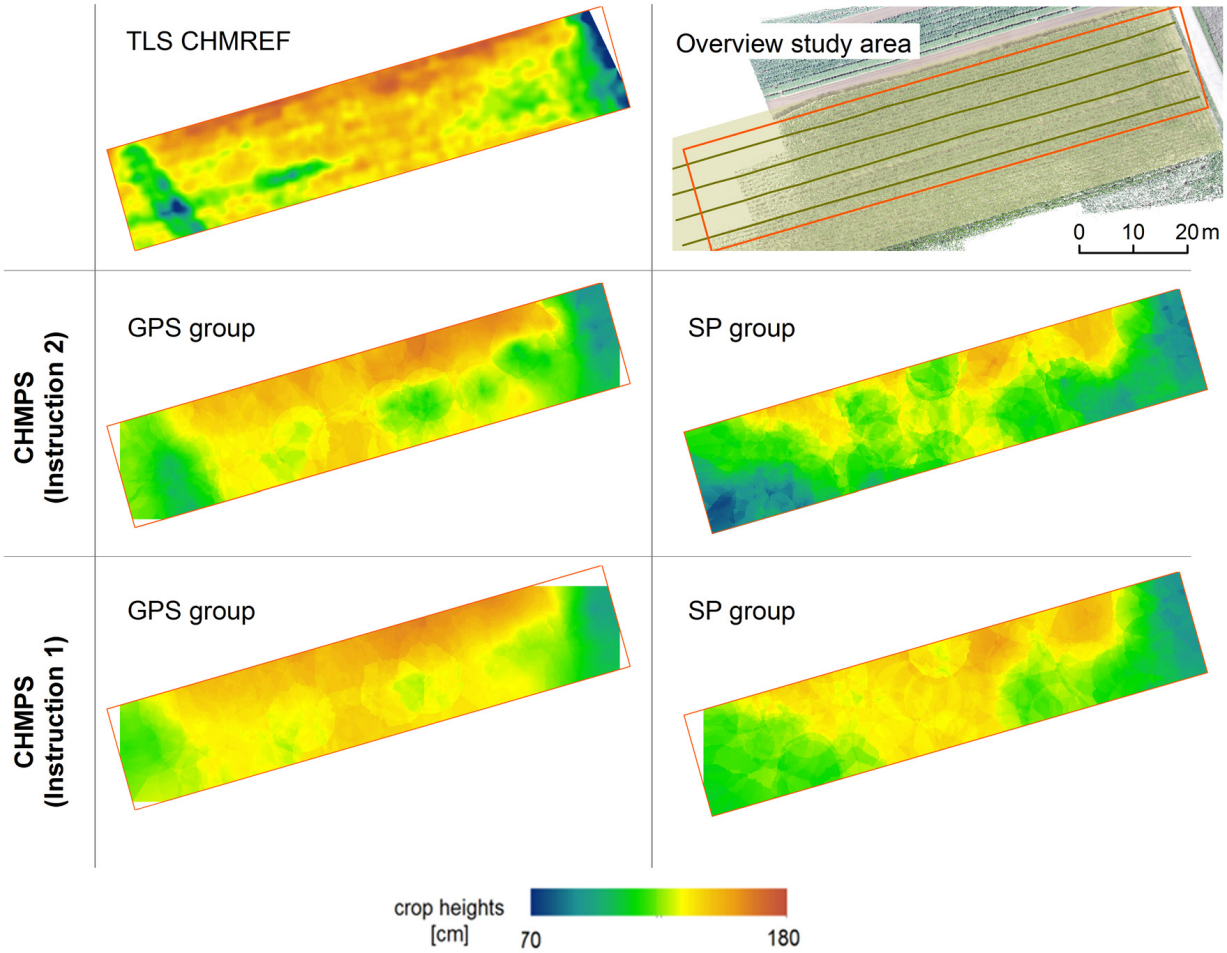
PS crop height models based on different instructions. Interpolated PS crop height models (CHMPS) based on Instruction 1+2 and on Instruction 1 only as well as the reference TLS CHM (CHMREF).

In order to analyze if PS-based crop height assessment benefits from a higher participation level, the participants are instructed in two different ways. I1 aims at gathering height values in a regular pattern, whereas I2 incorporates participants’ cognitive abilities to collect additional data in heterogeneous areas. In this section it is firstly described where participants capture additional data according to I2. Secondly, the influence on the resulting CHMPSs is discussed by comparing it with the CHMREF. 47 (GPS group) and 104 (SP group) additional crop height values according to I2 are captured. Additional measurements of the GPS group are mainly collected at the western end of the study area where the Roman street is located under the field (27 out of 47 measurements). In this area, high values of standard deviations (above 14 cm) representing heterogeneous plant heights in the TLS dataset are found. At the eastern end of the field, another heterogeneous area is identified but participants gathered only four additional crop height measurements based on I2. Although standard deviation in the middle of the study area is lower, 14 positions are marked as additional measurements within this area. This is mainly carried out by one single participant. In contrast to the findings based on the paper-based approach, the SP group collected GPS positions based on I1 all over the field. Furthermore, the percentage of additional measurements on the total number of measurements is higher (28%) compared to the GPS group (17%). The total number of measurements according to I2 ranges from 3 to 41 per participant. This study shows that correlation between PS and TLS heights is higher if height values are collected in a regular pattern based on I1 (R^2^ for I1: 0.63 [GPS], 0.24 [SP]; R^2^ for I2: 0.54 [GPS], 0.23 [SP]). However, I2 allows capturing information that cannot be reproduced when height values are collected at regular distance, for example, in case some distinct change in crop height is situated between two measurement points: As the width of the Roman street is about 5 m, lower height values might not be represented at all in this area in the case of I1, where data are gathered only every tenth step resulting in an estimated distance between measurements of 7 m (depending on the step size a participant takes). Thus, mean height difference between CHMREF and CHMPS is lower in the area of the Roman street (ca. 10 cm) if the additional measurements (according to I2) are included. In contrast, errors are introduced by gathering crop heights that are not representative for a certain area, thus influencing the CHM interpolation. This happens at the center of the study area where height variations are represented more correctly without additional measurements from I2. Detailed numbers on the comparison between the CHMPS rasters and CHMREF are given in Table 2, the spatial patterns are shown in Fig 8.

**Table 2:** Comparsion between interpolated PS crop height models and the reference TLS crop height model.

Difference raster for the study area / area of the Roman street	RMSE [cm]		SD [cm]		MEAN [cm]	
	I1	I2	I1	I2	I1	I2
**GPS group (study area)**	10.79	11.82	10.68	10.90	1.53	4.58
**GPS group (Roman street)**	22.59	11.20	15.09	10.24	-16.82	-4.55
**SP group (study area)**	15.36	25.02	8.69	15.14	12.67	19.92
**SP group (Roman street)**	17.76	12.40	13.54	11.2	-11.50	5.32

#### Image-based crop height assessment

The three data collection days resulted in 81 pictures of the marker bar. Eight of those pictures are manually labelled and used to build up the RF classifier. It classifies all pixels of the remaining 73 pictures into the classes: background, circle, reference area and marker bar. The region of interest is given by the bounding box of the circle probability which reduces potential misclassifications due to background objects with similar feature characteristic. Within this region, the vertical extent for the classes reference area and marker bar is determined in the postprocessed binary images. Quality varies between the pictures taken on the three days of data collection. This is caused by unfavorable weather conditions in terms of image acquisition on two of the days. On both days, pictures are captured in the later afternoon hours when light and weather conditions are poor. Nevertheless, in 57 out of 73 pictures crop height can be assessed. Image classification achieves over 80% average precision (class reference area: 83%, class marker bar: 87%); average recall accuracy is 84% for the reference area and 67% for the marker bar. 9 out of 73 pictures are too dark and blurry in order to extract the marker bar. Furthermore, in seven pictures either the marker bar or the reference area is misclassified.

Since the total height of the marker bar and the reference area are known, the height of the obscured part of the marker bar (corresponding to the crop height) can be calculated. For further evaluation, extracted PS crop heights captured on July 11 are analyzed in detail. On this day, 33 pictures are taken by three participants standing at nine different positions with a distance between 5 m and 28 m to the marker bar. In 26 out of those pictures, the marker bar is detected which leads to an average plant height of 135.63 cm (SD = 18.33 cm). The average height value resulting from the image-based approach differs less than 5 cm (relative error: 3.35%) from the CHMREF (140.33 cm) at the position of the marker bar. Hence, the proposed PS-based method leads to a similar accuracy of crop height measurements as also reported by Sritarapipat *et al*. [63]: For the crop heights of a rice paddy field extracted from images, an average relative error of 2.63% is reported. Compared to a fixed camera as used in [63], the PS approach offers more flexibility and allows assessing crop heights at a higher spatial resolution. It has to be noted that PS crop heights vary between the single positions from which the pictures are taken from on a decimeter-scale: As the maize is up to 180 cm high, not only plants in close neighborhood can obscure the marker bar but also plants standing in the line of the smartphone camera to the marker bar. Hence, in this experiment, the position of the photographer has an impact on the crop height measurements. This takes less into account if the presented method is applied for investigating fields with more homogeneous height patterns and lower plant heights.

The results of the RF classification approach can be further improved by a larger amount of training data which covers the weather-induced variability of the pictures better [76]. In this study, the participants’ input is used to restrict the area of interest for image classification. In addition, participants could also provide labels for training the machine learning algorithm. Hillen & Höfle [77] present a concept called *Geo-reCAPTCHA* with allows non-experts to create geographic information from Earth observation data: The resulting building polygons could be used to classify e.g. remote sensing data. Combining both crowdsourced labelling as well as active learning which allows iteratively fine-tuning the training dataset is a promising way for further improving object detection in images gathered via the PS approach [78]. A further advantage of the image-based method is that participants do not necessarily have to enter the crop field to assess plant heights. Photographs taken, for example, from a permanently installed marker bar can be captured from outside the field. This offers means for multi-temporal monitoring of crop heights in a simple way without any maintenance costs. For capturing crop height variations within a single field, M1 and M2 are better suited as presented in Section 5.2.1. Another photogrammetric pipeline increasingly used to derive 3D geodata is structure-from-motion and dense matching. The method provides the means to extract a detailed three-dimensional geometric representation of vegetation based on a random picture collection, captured for example with smartphone integrated cameras [79–81].

## Conclusions

This study presents a novel approach for PS-based crop height determination on level of individual fields by bringing together easy to use mobile data collection methods with state-of-the-art machine learning techniques. The gathered geodata on in-field crop height variations is suitable for spatial modelling or performing quantitative tasks e.g. site-specific agriculture. In the performed experiment, 19 participants collected 630 direct crop height measurements in total and took 81 pictures from a marker bar using smartphones and handheld GPS devices. Quality control of the PS crop height measurements is conducted with TLS data and results in R^2^ = 0.63 for the handheld GPS devices and R^2^ = 0.24 for the smartphone-based approach. The comparison between the reference TLS model (CHMREF) and the crop height model based on the PS (CHMPS) yields a RMSE of 10.45 cm (GPS group) and 14.60 cm (SP group).

This study suggests a strong relationship between crop heights estimated by symbols (human figures) and measured by ruler (GPS group: r_spearman_ = 0.91; SP group: r_spearman_ = 0.94). Accordingly, crop height determination can be performed without any additional equipment besides a smartphone which is an important prerequisite for lowering the barrier for participation (e.g. due to technical or educational reason). Furthermore, the potential of supervised machine learning for extracting quantitative height measurements from pictures taken by non-professional participants is shown (less than 5 cm height difference to the reference TLS height). Automatic processing of human observations based on photographs, involving computer vision and machine learning, offers new opportunities to extract information from a large amount of data [13].

By incorporating participants’ cognitive abilities, valuable information can be gathered. Collecting additional crop heights in heterogeneous areas based on the participants’ judgement (i.e. participants decide where to take height measurements (I2)) improves the quality of the PS crop height model e.g. in the area of the Roman street. However, as participants following I2 tend to gather extreme crop heights, the correlation between the PS and TLS crop height model based on I2 is weaker compared to I1 (i.e. height measurements are taken in regular distances). The presented mobile application based on the *ODK* is easy-to-use and provides instructions for the crop height assessment. Compared to the paper-based approach, mobile data collection saves time and human resources as no error-prone digitization of the gathered data is needed [28]. It can not only be used to gather in-field crop height variations but also to monitor, for example, plant heath, diseases and pests. In future, PS geodata combined with crop information obtained from other on-site or off-site sensors such as satellites or unmanned aerial systems (UAS) could be used for crop yield predictions at low-costs and on a small scale (individual fields). Smallholders are at the forefront of climate change impacts as crop failures reduce the per capita food availability of subsistence farmers. Further research is needed to understand multi-dimensional phenomena such as malnutrition and requires integrated approaches where data from different sensors are combined. Therein, PS can provide a valuable supplementary source of information based on contributions of local people.

## Supporting Information

S1 Dataset Participatory Sensing crop heights—SP group. Data was collected by 19 participants equipped with smartphones using human figures to estimate (M1) as well as a ruler to measure (M2) crop heights. Two different types of instructions were given to the participants for collecting in-field crop height variations: measure crop heights every tenth step in four predefined rows (I1) and add additional measurement positions in between if plants vary greatly in height (I2). Attributes: Latitude; Longitude; Instruction Type: I1 or I2; Crop Height—M1: crop heights estimated by the participants based on M1. Heights are categorized (1–10) representing the human figures that are marked at ten different height levels e.g. knee or head; Crop Height—M2 (cm): crop heights (in cm) measured by the participants based on M2.
(ZIP)

S2 Dataset Participatory Sensing crop heights—GPS group. Data was collected by 19 participants equipped with handheld GPS devices using human figures to estimate (M1) as well as a ruler to measure (M2) crop heights. Two different types of instructions were given to the participants for collecting in-field crop height variations: measure crop heights every tenth step in four predefined rows (I1) and add additional measurement positions in between if plants vary greatly in height (I2). Attributes: Latitude; Longitude; Instruction Type: I1 or I2; Crop Height—M1: crop heights estimated by the participants based on M1. Heights are categorized (1–10) representing the human figures that are marked at ten different height levels e.g. knee or head; Crop Height—M2 (cm): crop heights (in cm) measured by the participants based on M2.
(ZIP)

S1 Table Data collection forms for crop height assessment. Questions presented to the participants on their smartphone displays for crop height assessment.
(PDF)
