# Supplementary material for: 3D Participatory Sensing with Low-Cost Mobile Devices for Crop Height Assessment – A Comparison with Terrestrial Laser Scanning Data
Source: PLoS One. 2016 Apr 13;11(4):e0152839. doi: 10.1371/journal.pone.0152839 (PMC4830550; doi:10.1371/journal.pone.0152839)
Supplement: S1 Table — Questions presented to the participants on their smartphone displays for crop height assessment. (PDF) [file pone.0152839.s003.pdf]

S1 Table. Data Collection Forms for Crop Height Assessment

The following questions (translated from German to English) were presented to the participants on their smartphone displays using the ODK Collect app:

Direct Crop Height Measurements

Field	Question	Answer
Instructions	Please move on to the next location within the maize field following the instructions: measure crop heights every tenth step in the four predefined rows and add additional measurement positions in between if plants vary greatly in height. Then, start the plant height assessment.	
Method1	How high are the maize plants at your current position? Please choose the image which fits best:	Image 1-10, showing human figures marked at equal height intervals
Method2	Please measure the plant height at your current position using the ruler.	Plant height in cm
Location	Please record your current position.<br>*[Button: Record Position]*	XY-coordinate
Instruction1_2	Did you assess the plant height at your current position based on Instruction 1 (“measure crop heights every tenth step”) or Instruction 2 (“add an additional measurement position if plants vary greatly in height”)?	Instruction 1 or Instruction 2

Image-based Crop Height Measurements

Field	Question	Answer
Photo	Please take a picture of the maize field. Make sure that the smartphone camera is located above the plants focusing the marker bar.<br>After taking the picture, draw a circle around the marker bar on the touchscreen of your smartphones.<br>*[Button: Take a picture]*<br>*[Button: Edit picture]*	Picture including circle around the marker bar
Location	Enter the ID of your current position and proceed with the next position.	Location ID
